# Impact of ERAS compliance on the short-term outcomes for distal radius surgery: a single-center retrospective study

**DOI:** 10.1186/s13018-023-04178-6

**Published:** 2023-09-19

**Authors:** Mi Er A. Li Mu Mu Er Ti Zha, Zhi Jian Sun, Ting Li, Re Zi Ya Ai Mai Ti, Gang Fu, Dong Chen Yao, Xiang Yu

**Affiliations:** 1 Department of Orthopedic, People’s Hospital of Xinjiang Uygur Autonomous Region China, Urumqi, 830001 Xinjiang China; 2 Department of Orthopedic Trauma, Beijing Jishuitan Hospital, Capital Medical University, Beijing, 100035 China; 3 College of Traditional Uyghur Medicine, Xinjiang Medical University, Urumqi, 830017 Xinjiang China; 4 Department of Orthopedic, Fuzhou Second Hospital, Fuzhou, 350007 China

**Keywords:** Distal radius fracture, ERAS, Compliance, Distal radius surgery

## Abstract

**Background:**

Distal radius fractures (DRF) account for one in five bony injuries in both primary and secondary trauma care. Enhanced recovery after surgery (ERAS) has been adopted successfully to improve clinical outcomes in multiple surgical disciplines; however, no study has investigated the effect of different degrees of compliance with ERAS protocol on short-term outcomes following distal radius surgery. We aimed to analyze whether different degrees of compliance with the ERAS pathway are associated with clinical improvement following surgery for DRF.

**Methods:**

We retrospectively analyzed all consecutive patients with ERAS who underwent surgery for DRF at our department between May 2019 and October 2022. Their pre-, peri-, and post-operative compliance with the 22 elements of the ERAS program were assessed. We compared parameters between low- (< 68.1%) and high-compliance (> 68.1%) groups, including patient complications, total length of hospitalization, discharge time after surgery, hospital costs, time taken to return to preinjury level performance level, number of visual analogue scale (VAS) pain scores > 3 points during hospitalization, disabilities of the arm, shoulder and hand (DASH) scores. We performed multiple linear regression analyses to assess the impact of ERAS compliance on the postoperative function level (DASH scores).

**Results:**

No significant differences were detected between the high- and low-compliance groups with respect to demographics, including sex, age, body mass index (BMI), and comorbidities (*P* > 0.05). We observed significant differences between the high- and low-compliance groups in terms of the DASH score (32.25 ± 9.97 vs. 40.50 ± 15.65, *p* < 0.05) at 6 months postoperatively, the discharge time after surgery (2.45 ± 1.46 vs. 3.14 ± 1.50, *p* < 0.05), and number of times when the VAS pain score was > 3 points during hospitalization (0.88, [0.44, 1.31], *p* < 0.05). Our study demonstrated a significant negative association between ERAS compliance and the function level of patients postoperatively (DASH scores) when adjusted for age, comorbidity, sex, and BMI.

**Conclusions:**

This study provided a realistic evaluation and comparison of the ERAS protocol among patients with DRF and can guide clinical decision making. The ERAS protocol may improve outcomes after surgery, with high postoperative function levels and reduced pain and discharge time after surgery, without increased complication rates or hospital costs.

## Background

Distal radius fractures (DRF) are one of the most common fractures treated by orthopedic surgeons worldwide. The annual incidence of DRF was 643,000 in the US in 1998 [1, 2]. DRF is the most common adult orthopedic fracture in the UK, incurring an average expenditure of £1375.34 per-patient for surgical fixation with a volar locking plate [3]. In China, DRFs account for approximately 20% of emergency fractures and 75% of forearm fractures. The social health burden of DRF is greater in China than in other countries [4]. More than 50% of patients with DRF are employed; therefore, the impairment resulting from range of movement restriction, the duration of the sick leave, and the effects of DRF on quality of life have broad socio-economic consequences [5].

An enhanced recovery after surgery (ERAS) program is a multimodal, evidence-based approach that combines various measures from initial patient referral through discharge. ERAS protocols were introduced more than 20 years ago by Henrik Kehlet, involving a multidisciplinary team comprising orthopedic surgeons, nursing staff, anesthesiologists, internists, physiotherapists, and nutritionists [6]. The ERAS pathway has been developed to reduce the negative effects of surgical stress. Additional key issues in ERAS programs in orthopedic surgery include effective pain treatment and management, which undoubtedly influence early hospital discharge and fast recovery at home [7]. However, some studies evaluating the discharge procedure and patients’ experiences after discharge demonstrated that early discharge, particularly in older adults, may be stressful in terms of managing daily life and rehabilitation [8]. This type of ERAS pathway has clear advantages and represents the standard of care across several institutions; nonetheless, the clinical effectiveness of ERAS procedures has not been established for all areas of orthopedic practice so significant work and research are required [9]. Moreover, ERAS pathways are continuously improving. Thus, continuous evidence-based revisions of ERAS use in different areas of orthopedic practice are mandatory to update orthopedic surgeons and staff on the use of these pathways and their potential advantages over traditional protocols in terms of safety and efficacy. Previous studies have reported higher levels of satisfaction among patients undergoing orthopedic surgery with the application of the ERAS protocol, compared with the traditional healthcare pathway [10]. However, ERAS is applied late in orthopedic trauma, and the ERAS pathways have been insufficiently developed for patients with fractures, except for older adults with hip fractures [11]. The effects of ERAS on short-term postoperative outcomes have not yet been investigated among other patients with fractures. Our previous research showed better functional recovery among patients with DRF when the ERAS protocol was applied than when it was not [12].

The indications for DRF surgical interventions are well defined; however, the standard perioperative protocol based on the ERAS program remains unpopular worldwide. Based on the updated ERAS guidelines for other fractures, the peri-operative ERAS pathways for DRF have been optimized in China, focusing on short perioperative fasting duration, minimally invasive surgical approaches, minimizing the use of drains and tubes, multimodal pain control, and early mobilization [3].

Moreover, research on patient compliance with ERAS is limited. Thus, we aimed to compare the demographic characteristics and outcome measures between low and high ERAS compliance groups undergoing open reduction and internal fixation for DRF. We also explored the associations between ERAS compliance and postoperative function (disabilities of the arm, shoulder and hand, DASH scores), length of hospitalization (LOS), hospital costs, complications, and time to achieve pre-injury level of functional activity.

## Methods

### Study design and participants

This retrospective, single center clinical controlled study was approved by the ethics committee of Beijing Jishuitan Hospital, China (JST202101-14). The entire ERAS protocol was implemented by a multidisciplinary ERAS team. Patients with DRF were enrolled in the study between May 2019 and October 2022.

Written informed consent was obtained from the patients before surgery. The inclusion criteria were as follows: (1) age ≥ 14 years and fractures < 3 weeks old; (2) a primary diagnosis of DRF, according to the following surgical indications for operative fixation of the American Academy of Orthopedic Surgeons clinical practice guidelines: post-reduction radial shortening of 3 mm, dorsal tilt of 10°, or intra-articular displacement or step-off of 2 mm [13]; (3) no serious systemic disease with a preoperative American Society of Anesthesiologists classification I-II; and (4) voluntary and informed consent to undergo surgery. The exclusion criteria were as follows: (1) DRF combined with other fractures (combined with ulnar styloid fracture or distal ulnar fracture requiring ulnar fixation); (2) old DRFs (> 3 weeks); (3) open fractures (excluding Gustilo type I); (4) severe peripheral neuropathy; (4) a diagnosis of gastric emptying disorders; (5) alcohol dependence or a history of drug abuse; (6) breastfeeding or pregnancy; (7) a history of allergy to multiple drugs; and (8) patients lost to follow-up.

Data were collected by a trained surgeon and verified before entry into an internet-based electronic case record form designed specifically for this study.

### ERAS pathway

The ERAS pathway was a standard perioperative protocol focusing primarily on pain management, perioperative fasting, and avoiding drains and urinary tubes [3]. For pain management, we prescribed oral multimodal analgesics preoperatively and opioid-free nerve block-based anesthesia postoperatively. We followed a standard anesthesia protocol for a painless procedure. For perioperative fasting, we minimized the fasting time to the greatest extent possible, with low risks of pulmonary aspiration to reduce perioperative stress [14]. For tube management, the urinary catheterization rate was reduced and meticulous hemostasis was achieved intraoperatively to avoid wound drains [15].

Multimodal analgesia in the ERAS protocol setting involves combinations of nonsteroidal anti-inflammatory drugs (NSAIDs) and paracetamol, which all target different pain receptors, along with ultrasound-guided regional anesthesia. NSAIDs were routinely administered before surgeries, and peripheral nerve blocks were administered if necessary. During ultrasound-guided regional anesthesia of the infraclavicular brachial plexus, 20 cc of 0.5% ropivacaine was gradually injected to the target point. Clinicians were able to provide additional pain medications to patients during the surgery, as clinically indicated, if the patient’s pain was not controlled by the block alone.

### Process of care metrics

According to the consensus on the optimal treatment of DRFs [4], we evaluated 22 ERAS perioperative care items using the following process of care metrics. For perioperative fasting management, we evaluated the perioperative fasting period for solids and liquids. For perioperative time management, we evaluated the elapsed time between admission and surgery, discharge time after operation, and LOS. For perioperative nonsurgical management, we assessed the use of regional anesthesia, close reduction, and nonsteroidal anti-inflammatory drugs, the prevention of postoperative nausea and vomiting, intraoperative capacity management, patient-controlled analgesia, antiemetics, urinary catheterization, wound drains, and postoperative rehabilitation exercise (Table 1).

**Table 1:** ERAS pathway of distal radial fractures

Care process	Description
*Preoperative items*	
Patient education	The concept of ERAS, including shortening the fasting time, pain education, smoking, and drinking
Reduction and fixation	Reduction under anesthesia for displaced distal radius fractures
Preoperative pain control	Oral NSAIDs to prevent hyperalgesia
Nutritional Assessment	Complete nutritional assessment for patients within 24 h after admission, and nutritional intervention for patients with nutritional risks
Blood glucose management	Avoid hypoglycemia while controlling hyperglycemia. Blood glucose is maintained at levels between 7.8 and 10 mmol/L
Soft tissue swelling treatment	Various physical methods are administered to reduce swelling during the perioperative period
Treatment of skin tension blisters	Wait for the blister to absorb, cover with a dressing, and absorb the blister liquid
Preoperative dietary management	Clear liquids allowed up to 2 h and solids up to 6 h before anesthesia
*Intraoperative items*	
Pre-operative urinary catheterization	No routine urinary catheterization
Antibiotic prophylaxis	Antibiotics within 30 min before incision
Tourniquet management	Shorter timing of tourniquet application and lower pressure of the tourniquet
Standard anesthesia protocol	Brachial plexus block recommended for distal radial fracture
Intraoperative blood pressure control	Maintain intraoperative blood pressure to reduce intraoperative tourniquet pressure and intraoperative bleeding
Fluid management	Avoid excess hypertonic fluid, particularly sodium-containing fluid
Prevention of hypothermia	Routine body temperature monitoring and active warming devices
Avoidance of drains	Meticulous hemostasis and no wound drains
*Postoperative item*	
Early feeding	Gradual oral intake of liquid sand solids after recovery from anesthesia
Early mobilization	Early mobilization within 24 h
Multimodal analgesia	Multimodal opioid-free analgesia based on nerve block
Prevention of postoperative PONV	For high-risk patients, medication can be administered to stop vomiting
Functional exercise	Perform functional exercises as early as possible after surgery
Clinical follow-up	Assigned feasible discharge criteria and follow up patients for at least 6 months

*NSAID* non-steroidal anti-inflammatory drugs, *PONV* postoperative nausea and vomiting, *ERAS* enhanced recovery after surgery

The compliance rate was calculated as follows:

ERAS compliance rate (%) = the number of perioperative measures performed as required/total treatment measures × 100% [16].

For example, if a patient completed 17 out of 22 items, compliance rate (%) = (17/22) × 100% = 77.27%.

### Outcome metrics

The primary outcome was the DASH score reflecting patient function level at 6 months postoperatively after treatment completion [17]. The secondary outcomes included the elapsed time between admission and surgery, discharge time after operation, hospitalization time, postoperative complications, costs of treatment, and number of times when the visual analogue scale (VAS) pain score was > 3 points during hospitalization. The postoperative complications included the loss of reduction, screw breakage assessed by routine evaluation of postoperative radiographs, respiratory infections, and time to achieve pre-injury level of functional activity. DASH scores were recorded at 6 months after surgery. Because the potential influence of other factors may affect postoperative recovery, we selected appropriate covariates, such as age, sex, BMI, and comorbidity, based on previous literature [18, 19].

### Statistical analysis

All data analyses were performed using R software (version 4.0.3; https://www.R-project.org) and Stata (version 16 http://www.stata.com). Data were considered statistically significant at *P* < 0.05. This study included participants with complete data in the database. Those with missing data were excluded from the analysis. Continuous variables are described using means and standard deviations (SD) for the baseline characteristics, whereas categorical variables are described using a chi-square test. The regression coefficients (*β*) and 95% confidence intervals (CI) were calculated from the multiple linear regression equations to analyze the association between ERAS compliance and DASH scores. Model 1 regression models did not adjust for the covariates; model 2 adjusted for three covariates, namely, sex, age, and BMI; and model 3 adjusted for all covariates (*P* < 0.05 indicated statistical significance).

## Results

### Demographic characteristics

We collected the data of 88 patients with DRFs treated using the ERAS perioperative protocol. Based on a 68.1% median level of ERAS compliance rate, we divided the patients into the high- (compliance rate ≥ 68.1%) and low-compliance (compliance rate < 68.1%) groups. Table 2 summarizes patient demographic characteristics.

**Table 2:** Demographic characteristics

Variables	Lower compliance (*n* = 40)	High compliance (*n* = 48)	Standardize diff	*P* value
Age (y)	52.50 ± 13.74	48.42 ± 17.74	0.26 (− 0.16, 0.68)	0.238
Sex (male, %)			0.30 (− 0.12, 0.73)	0.170
Male	13 (33.33%)	23 (47.92%)		
BMI (kg/m^2^)	25.29 ± 4.01	25.49 ± 3.65	0.05 (− 0.37, 0.47)	0.807
Comorbidity (yes, %)	12 (30.00%)	10 (20.83%)	0.21 (− 0.21, 0.63)	0.323
ERAS compliance (%)	61.89 ± 3.70	72.94 ± 4.15	2.81 (2.22, 3.40)	< 0.001

*BMI* body mass index, *ERAS* enhanced recovery after surgery

### ERAS process

Most process metrics of the included patients demonstrated significant differences between the low- (*n* = 40) and high-compliance (*n* = 48) groups. All the patients completed their follow-up at 6 months postoperatively, and there were no complications such as the loss of reduction or screw breakage assessed by routine evaluation of postoperative radiographs. For perioperative management, no significant between-group differences were observed in LOS (days) (7.15 ± 2.54 vs. 7.19 ± 2.61, *P* > 0.05) and the elapsed time between admission and surgery (day) (3.95 ± 1.81 vs. 4.12 ± 1.68, *P* > 0.05). The discharge time after surgery (days) (2.45 ± 1.46 vs. 3.14 ± 1.50, *P* < 0.05) was significantly shorter in the high-compliance group than in the low-compliance group. We observed no significant between-group differences in medical costs (¥) (58.654.31 ± 37.160.82 vs. 53.179.78 ± 12.577.65, *P* > 0.05), time to achieve pre-injury level of functional activity (days) (88.73 ± 43.21 vs. 90.00 ± 37.76, *P* > 0.05), and complications (n, %) after surgery (SD, 0.45 [0.02, 0.87], *P* = 0.069). The high-compliance group demonstrated lower DASH scores at 6 months postoperatively (PO6M) (32.25 ± 9.97 vs. 40.50 ± 15.65, *P* = 0.004) and fewer VAS pain scores of > 3 points (SD, 0.88 [0.44, 1.31], *P* = 0.003) than the low-compliance group (Table 3).

**Table 3:** Postoperative indicators between the groups

Variables	Low compliance (*n* = 40)	High compliance (*n* = 48)	Standardize diff	*P* value
LOS (days)	7.15 ± 2.54	7.19 ± 2.61	0.30 (− 0.12, 0.73)	0.946
Elapsed time between admission and surgery (days)	3.95 ± 1.81	4.12 ± 1.68	0.10 (− 0.32, 0.52)	0.639
Discharge time after surgery	3.14 ± 1.50	2.45 ± 1.46	0.47 (0.05, 0.90)	**0.007**
Medical costs (¥)	53,179.78 ± 12,577.65	58,654.31 ± 37,160.82	0.20 (− 0.22, 0.62)	0.376
Time back to pre-injury level of function (days)	90.00 ± 37.76	88.73 ± 43.21	0.03 (− 0.39, 0.45)	0.885
Complications (*n*, %)	2 (5.00%)	2 (4.17%)	0.45 (0.02, 0.87)	0.069
Number of times when the VAS pain score was > 3 points			0.88 (0.44, 1.31)	**0.003**
1	10 (25.00%)	30 (62.50%)		
2	17(42.50%)	11 (22.92%)		
3	10 (25.00%)	7 (14.58%)		
4	3 (7.50%)	0 (0.00%)		
DASH score	40.50 ± 15.65	32.25 ± 9.97	0.63 (0.20, 1.06)	**0.004**

Bold: *P* < 0.05 indicated statistical significance

*DASH* disabilities of the arm, shoulder and hand, *LOS* length of hospitalization, *VAS* visual analogue scale

**Fig. 1:**
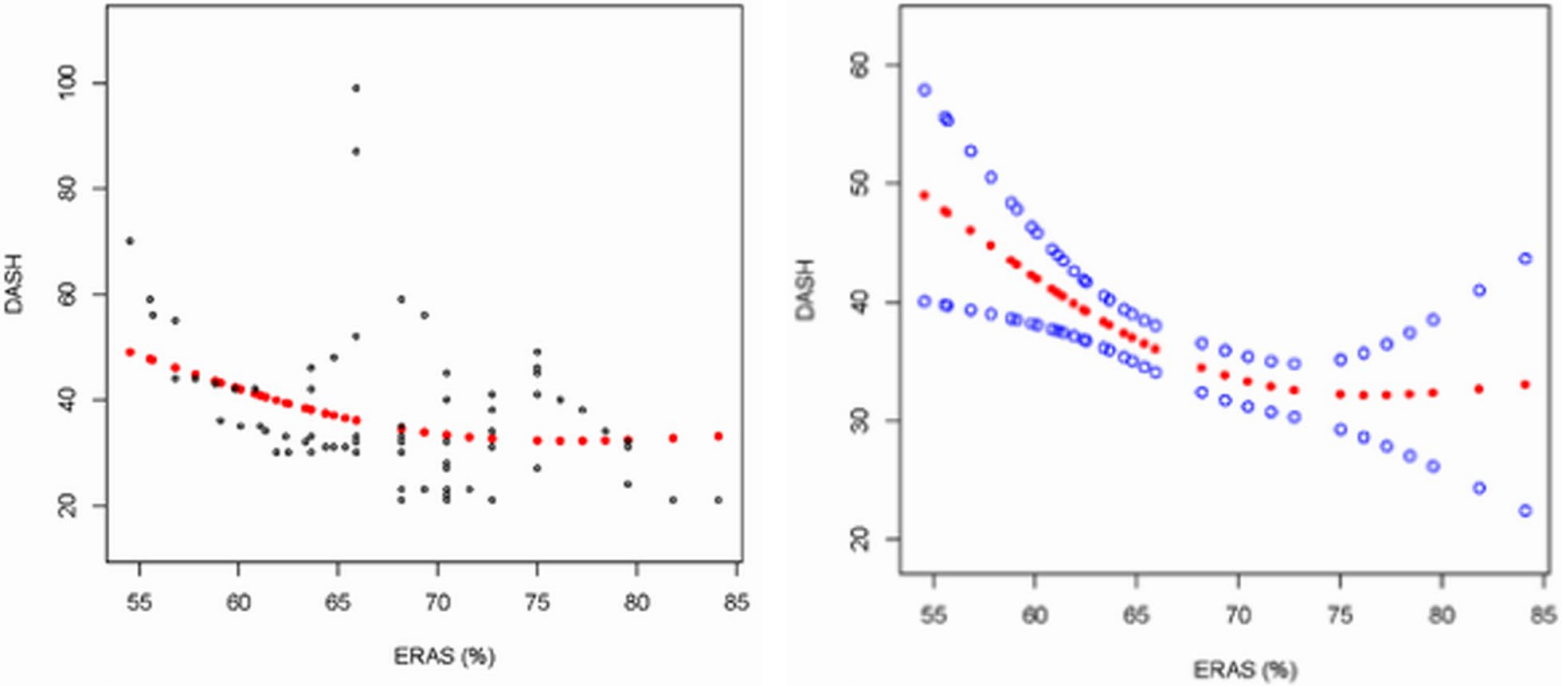
Relationship between ERAS compliance (%) and DASH score. (**Right**) Each black dot represents a sample. (**Left**) Red dots represent smoothed curves between the variables, and the blue bands represents the 95% confidence interval of the fitted curve (adjusted for the age, sex, BMI, and comorbidity)

To examine the association between ERAS compliance and DASH scores at PO6M, we performed a multivariable linear regression analysis. The model was adjusted for sex, age, comorbidity, and BMI. The results presented in the three models demonstrated a negative association between ERAS compliance and DASH scores. After adjusting for all covariates (Model 3), ERAS compliance was negatively associated with the DASH scores (*β* =  − 0.58; 95% CI: − 1.01, − 0.14; *P* = 0.0118). Figure 1 and Table 4 depict the smoothed curves of ERAS compliance and DASH scores.

**Table 4:** Associations between ERAS compliance and DASH scores

	Model 1 *β* (95% CI) *p* value	Model 2 *β* (95% CI) *p* value	Model 3 *β* (95% CI) *p* value
ERAS compliance (%)	− 0.62 (− 1.04, − 0.20) 0.0048	− 0.61 (− 1.04, − 0.17) 0.0074	− 0.58 (− 1.01, − 0.14) 0.0118
*P* trend	< 0.05	< 0.05	< 0.05

Model 1: No covariates were adjusted for

Model 2: Age, sex, and BMI were adjusted for

Model 3: Age, sex, BMI, and comorbidity were adjusted for

*BMI* body mass index, *DASH* disabilities of the arm, shoulder and hand, *ERAS* enhanced recovery after surgery

## Discussion

ERAS protocols have been adopted by various surgical departments. Most of the studies examining the outcomes of ERAS assessed functional improvement after surgery, such as LOS, hospitalization expense, and complication rates between a non-ERAS group and ERAS group [20]. However, few studies have explored ERAS compliance. This retrospective study, which compared the short-term postoperative outcomes of patients with DRF and different degrees of compliance with ERAS, demonstrated that participants in the high-compliance group recovered better, with improved DASH scores, than patients in the low-compliance group, with less time to discharge following surgery and fewer VAS scores of > 3 points during hospitalization.

Pain management, perioperative fasting, and avoiding tubes are the three primary constituents of the ERAS protocol. We prescribed multimodal analgesia and minimal fasting time and strived to achieve meticulous hemostasis to reduce the use of wound drains and early functional exercise. Previously, patients demonstrated satisfactory adherence to the ERAS protocol and those in the ERAS group exhibited a higher satisfaction level at discharge and at 6 months postoperatively, compared with those in the traditional healthcare group [8, 17, 18]. This may be a result of the ERAS protocol and may also reduce the incidence of depression and anxiety at a severity of moderate or above and the degree of depression and anxiety [21].

Mandated diagnosis-related groups (DRGs) have been used worldwide to curb resource demands with cost-saving implications; therefore, the application of the ERAS protocol may significantly contribute to the implementation of DRGs [22, 23].

The possible mechanisms and advantages of the protocol are as follows: first, individual conditions of participation are considered, and a patient-center approach will optimize the management [24]. Second, appropriate reduction and fixation under emergent anesthesia will facilitate early mobility and pain relief [25].

Moreover, the selection of anesthesia, such as peripheral nerve blocks, may improve postoperative pain relief [26]. In addition, preoperative education can comfort patients; nutritional intervention can assist with survival during the perioperative period, which could improve patient prognosis and satisfaction [25]. The ERAS protocol emphasizes the need for education and benefits patients who abandon unhealthy habits, such as smoking [27]. Patients with opioid use disorders tend to experience increased odds of complications, extended hospitalization, nonhome discharge, and higher total costs [28]. However, the implementation of the ERAS protocol could improve patient pain care and reduce opioid use, which was confirmed in the present study. Patients in the high-compliance group demonstrated a significantly shorter discharge time after surgery than those in the low-compliance group (*P* < 0.05). However, we identified no significant difference in the total medical costs, LOS, and the elapse time between admission and surgery, contradicting previous evidence that the ERAS protocol could shorten the LOS [9, 29] and reduce medical costs [30, 31]. This finding was attributed to the admission of patients over the weekend (36 patients, 41% of cases) and to the fact that tension blisters around the wrist joint may delay the time to surgery (25 patients corresponding to 28.4% of cases, presented with swelling and blisters around the wrist. However, the discharge time after surgery in the high-compliance group was significantly shorter than that in the low-compliance group. Thus, further research is necessary to confirm our findings.

Within an optimized and clear ERAS protocol, selected high-risk patients may benefit from accelerated recovery, with lower risk of complications, readmissions, and morbidity, without increasing the rate of complications and medical costs. Consistent with our previous research, the ERAS protocol can accelerate rehabilitation in patients with osteoporotic vertebral compression fracture [32].

ERAS programs often require a multidisciplinary team providing a multimodal approach to resolving issues that delay recovery and lead to complications. The protocol may consist of several elements; however, they have a common aim, i.e., minimizing stress and improving stress response [33]. In orthopedic surgery, clinical ERAS studies have primarily focused on arthroplasty [34, 35]. Few studies have focused on ERAS in orthopedic trauma, except for the only well-developed ERAS pathway used for hip fractures in older adults [36]. Considering the high incidence of orthopedic trauma, it is important to establish a scientific, evidence-based ERAS protocol for common fractures. Therefore, we assessed the value of the ERAS principles in common DRF.

This study had several strengths. First, we enrolled numerous patients with a similar fracture type. Second, unlike most studies involving ERAS principles in patients with fractures, we conducted a single-center retrospective cohort study, significantly reducing the risk of information bias. Third, this novel study determined the application of ERAS compliance in common fractures. Moreover, covariates that may affect postoperative recovery were used in multiple regression equation models to assess the association between ERAS compliance and DASH scores. Furthermore, our primary outcome was patient function level at 6 months postoperatively. It is a subjective, patient-oriented evaluation, reflecting the perioperative experience and short-term functional outcomes of the patients.

Nevertheless, the present study had some limitations. The present data were only generated from a single center and a follow up was only for 6 months, which did not enable us to draw definitive conclusions. Therefore, future prospective multicentric randomized controlled trials with larger cohorts and long-term follow-ups based on large-scale population are still required to understand the benefits of the ERAS protocol among patients with DRF. Second, we used the DASH scores to better describe the postoperative feelings of patients. The Gartland-Werley score system, which includes certain characteristics and strengths, can also be used to evaluate hand and wrist function in DRFs [37].

## Conclusions

In conclusion, the application of ERAS compliance protocol resulted in better short-term functional recovery with improved DASH scores among patients with DRF. In addition, the protocol reduced discharge time after surgery and the number of times with a VAS pain score > 3 points during hospitalization; thus, it may improve outcomes after surgery. A broader implementation of the ERAS protocol may identify further advantages of the protocol.

## Data Availability

The datasets used and/or analyzed during the current study are available from the corresponding author on reasonable request.

## Abbreviations

DASH: Disabilities of the arm, shoulder and hand
DRF: Distal radius fractures
ERAS: Enhanced recovery after surgery
LOS: Length of hospitalization
NSAID: Non-steroidal anti-inflammatory drugs
PONV: Postoperative nausea and vomiting
VAS: Visual analogue scale
PO6M: 6 Months postoperatively
